# † *Camelosphecia* gen. nov., lost ant-wasp intermediates from the mid-Cretaceous (Hymenoptera, Formicoidea)

**DOI:** 10.3897/zookeys.1005.57629

**Published:** 2020-12-18

**Authors:** Brendon E. Boudinot, Vincent Perrichot, Júlio C. M. Chaul

**Affiliations:** 1 Department of Entomology & Nematology, University of California, Davis, One Shields Ave, Davis, CA 95616, USA University of California Davis United States of America; 2 Friedrich-Schiller-Universität Jena, Institut für Zoologie und Evolutionsforschung, 1 Erberstraße, 07743 Jena, Thüringen, Germany Friedrich-Schiller-Universität Jena Germany; 3 Univ. Rennes, CNRS, Géosciences – UMR 6118, F-35000, Rennes, France Univ. Rennes, CNRS Rennes France; 4 Pós-Graduação em Ecologia, Departamento de Biologia Geral, Universidade Federal do Viçosa, 36570-900, Viçosa, MG, Brazil Universidade Federal do Viçosa Viçosa Brazil

**Keywords:** classification, identification key, Mesozoic revision, morphology, paleontology, phylogeny, taxonomic synopsis

## Abstract

Fossils provide primary material evidence for the pattern and timing of evolution. The newly discovered “beast ants” from mid-Cretaceous Burmite, †*Camelosphecia***gen. nov.**, display an exceptional combination of plesiomorphies, including absence of the metapleural gland, and a series of unique apomorphies. Females and males, represented by †*C. fossor***sp. nov.** and †*C. venator***sp. nov.**, differ in a number of features which suggest distinct sexual biologies. Combined-evidence phylogenetic analysis recovers †*Camelosphecia* and †*Camelomecia* as a clade which forms the extinct sister group of the Formicidae. Notably, these genera are only known from alate males and females; workers, if present, have yet to be recovered. Based on ongoing study of the total Aculeata informed by the beast ant genera, we provide a brief diagnosis of the Formicoidea. We also provide the first comprehensive key to the major groupings of Mesozoic Formicoidea, alongside a synoptic classification in which †Zigrasimeciinae**stat. nov.** and †*Myanmyrma maraudera***comb. nov.** are recognized. Finally, a brief diagnosis of the Formicoidea is outlined.

## Introduction

Ants are the dominant lineage of surface-dwelling eusocial insects, filling tropical canopies, permeating leaf litter, and shaping ecosystems through predation, granivory, herbivory, and a plethora of other means (Hölldobler and Wilson 1990). The fossil record of ants spans more than 100 million years of geological time, with hundreds of species attributed to modern taxa in the Cenozoic (e.g., Mayr 1868; Wheeler 1915; LaPolla et al. 2013), and a Mesozoic fauna with over 50 known species (e.g., Barden 2017; Barden and Engel 2019). It is the Mesozoic fossils which have imparted the deepest insight into ant evolution, from the first description of a stem-ant species which refined our knowledge of the ant ground plan (Wilson et al. 1967a, b), to the first Cretaceous crown ants (Grimaldi and Agosti 2000, McKellar et al. 2013a) which have informed our chronological estimates of ant origins (e.g., Brady et al. 2006; Moreau et al. 2006; Borowiec et al. 2019). Some of the most remarkable specimens, however, have been representatives of a lost fauna of highly modified top predators, including the so-called “hell ants” (†Haidomyrmecinae, e.g., Barden et al. 2020; Perrichot et al. 2020) and “iron-maiden ants” (†Zigrasimeciinae stat. nov., e.g., Cao et al. 2020).

Here, we report and describe a new genus of singularly bizarre “beast ants” from mid-Cretaceous Burmite, †*Camelosphecia* gen. nov., which combines novel autapomorphies with critical plesiomorphies of the ancestral Aculeata, such as absence of the metapleural gland. Based on phylogenetic analysis of genomic and morphological data spanning the total Aculeata (Boudinot et al. 2020a), we have found that †*Camelosphecia* is closely related to the hitherto unplaced genus †*Camelomecia*, with the two forming a clade which is sister to the Formicidae. The †*Camelomecia* clade informs the polarization of character states throughout the total Formicidae and foreshadows the discovery of other illuminating fossils. In order to contextualize the description of this new genus, we synthesize the systematics and morphology of the Cretaceous ant fauna as a synoptic classification and provide the first key to the major groups of Formicoidea.

## Materials and methods

Morphological observations which formed the foundation of this study were made via comparison of dry-mounted extant specimens, hand-cut amber fossils, digital photomicrographs from several sources (particularly AntWeb.org), and graphic representations in the literature. Several microscopes were used for examining physical material, with primary reliance on a Leica MZ 12A with fluorescent and fiber-optic lighting. Photomicrographs were taken with a variety of equipment, including a JVC KY-F57U digital camera mounted on a Leica MZ 16A microscope, a Canon 1100D digital camera mounted on a Leica S8APO, and a Olympus BX60 microscope equipped with fluorescent light source, with resultant z-stacks processed via Auto-Montage Pro (Synoptics Ltd. Cambridge England) or Zerene Stacker (Zerene Systems LLC). Figures were processed with Adobe Photoshop 2020 and Illustrator 2020 (Adobe Systems Inc. California, U.S.A.). Measurements were taken from photomicrographs using Photoshop.

### Terminology

Morphological terminology follows Richter et al. (2020) for the cranium and its appendages, Boudinot (2015) for the mesosoma and legs, Brown and Nutting (1950) for the abscissae of the wing veins, Bolton (1990) and Keller (2011) for the metasoma, and Harris (1979) for surface sculpture. However, we have found that it was necessary to further differentiate among structures and their corresponding terms based on further study of development and anatomy. In the present work, we refer to the “pronotal rim” of †*Camelosphecia*, which is the carina which margins the disc of the pronotum ventrally, and which should not be confused with the anteromedian lobe (“neck”) of the pronotum. We also refer to the “lateral pronotal lobes”, which correspond to the paired posterolateral extensions of the pronotum which conceal the mesothoracic spiracle, as is used for Apoidea.

Generally, the antenna is considered to comprise three segments, the scape, pedicel, and multi-annulate flagellum (Snodgrass 1935; Goulet and Huber 1993). However, we distinguish between the *radicle* and the *scape* based on molecular developmental study demonstrating correspondence between the radicle with the coxa plus trochanter, the scape with the femur, the pedicel with the tibia, and the multi-annulate flagellum with the tarsus (Toegel et al. 2009). The subdivisions of the radicle are recognized as the “bulbus” and “bulbus neck” in the ant literature (e.g., Keller 2011), and we employ those terms here where appropriate. We do note that Toegel et al. (2009) mislabeled the radicle as the antennifer, which is the condyle of the cranium articulating with the antenna (Snodgrass 1935).

Regarding setation, “hairs” are properly considered as mechanosensory *sensilla trichodea* (Chapman 2012), with three forms commonly expressed among Hymenoptera: *s. trichodea filiformis*, or setae in the strict sense, i.e., non-tapering or very narrowly-tapering setae; *s. trichodea chaetiformis*, or chaetae (“traction setae”), i.e., tapering or conical setae; and *s. trichodea psammochaetis*, or psammochaetae (“fossorial chaetae”), i.e., large, flattened, and often longitudinally-grooved setae. Regardless of form, *sensilla trichodea* on contact surfaces such as on the tibiae and tarsi are functionally significant as they provide traction in addition to sensory information (see references in Boudinot et al. 2020b). Chaetae occur most often on the tarsi, although they may be present on the tibiae, femora, and on the perioral sclerites. For example, the “traction setae” on the meso- and metatibiae of *Cryptopone*, *Centromyrmex*, and other Ponerinae (e.g., Bolton and Fisher 2008; Schmidt and Shattuck 2014) are chaetae used for gaining traction in soil tunnels, and the “spicules” or “clypeal” and “labral pegs” of various Leptanillinae, Amblyoponinae, and stem ants (e.g., Boudinot 2015) are short, stout chaetae. With respect to the labral chaetae of taxa treated in this study, we clarify that the terms “above”, “below”, dorsal, and ventral should be avoided, as the chaetae are only expressed on the aboral face of the labrum, and which have locations that are described by the lateromedial and proximodistal coordinate axes on the sclerite. Finally, we note that psammochaetae are widespread in fossorial Aculeata, often occurring in stereotyped positions on the legs of Tiphioidea, Thynnoidea, Pompilidae, Mutillidae, Bradynobaenidae, Scoliidae, and spheciform Apoidea.

### Measurement abbreviations (all in mm):

**A1L** Antennomere I length. Length of the main body of the scape, excluding the radicle.

**A2L** Antennomere II length. Length of the main body of the pedicel.

**A3L** Antennomere III length. Length of the first flagellar antennomere.

**CL** Cranium length. As measured in profile view, the length of head from posteriormost head margin as would be observed in full-face view to anteriormost discernible margin.

**CW** Clypeus width. Maximum measurable width of the clypeus in the most full-face view perspective achievable.

**EL** Eye length. Maximum measurable length of the compound eye.

**HL** Head length. As measured in profile view, the length of the head from the posteriormost head margin as for CL to the apicalmost point of the mandibles.

**HW** Head width. Maximum width of the cranium as measured in dorsal view.

**LW** Lateral ocellus width. Maximum measurable width of one of the lateral ocelli; note: lateral ocellus length measured for male due to specimen orientation.

**MsL** Mesoscutellum length. Maximum length of the mesoscutellum in dorsal view.

**MsW** Mesoscutellum width. Maximum width of the mesoscutellum in dorsal view.

**MtL** Mesoscutum length. Maximum length of the mesoscutum in dorsal view.

**MtW** Mesoscutum width. Maximum width of the mesoscutum in dorsal view.

**MW** Median ocellus width. Maximum measurable width of the median ocellus.

**PnL** Pronotal medial length. Length of the pronotum along its midline in dorsal view (note: measured in profile view for the male diagonally from the anterior pronotal margin to the posteriormost point of the medial margin).

**PnLm** Pronotal maximal length. Maximum length of the pronotum as measured between the pronotal lobes to the anteriormost discernible point (note: measured in profile view for the male diagonally from the anterior pronotal margin to the posteriormost point of the lateral pronotal lobe).

**PnW** Pronotal width: Maximum measurable width of the pronotum in dorsal view.

**PtL** Petiole length. Maximum discernible length of the petiole.

**WL** Mesosoma length. Diagonal length of the mesosoma as measured from the anteriormost pronotal angle to the posteriormost apex of the propodeal projection in dorsal view.

**WLa** Mesosoma length, alternative. Diagonal length of mesosoma as measured in profile view from the pronotal inflection to the posteriormost point of the propodeal projection.

**VBL** Vertexal bulge length, male specimen. As measured in profile view and in the same line as CL, the length of the vertexal bulge of the male from the occipital carina to the dorsal point of the cranium, excluding the ocelli.

### Repositories of material examined for specific results here reported

**AMNH**American Museum of Natural History, New York, New York, USA;

**BEBC**Brendon E. Boudinot collection, University of California, Davis, CA, USA. and Friedrich-Schiller-Universität Jena, Thüringen, Germany;

**CNUC**Key Lab of Insect Evolution and Environmental Changes, College of Life Sciences, Capital Normal University, Beijing, China;

**DZUP**Coleção Entomológica Padre Jesus Santiago Moure, Universidade Federal do Paraná, Curitiba, Paraná, Brazil;

**JCMC**Júlio C. M. Chaul collection, Universidade Federal de Viçosa, Minas Gerais, Brazil;

**NIGP**Nanjing Institute of Geology and Palaeontology, Chinese Academy of Sciences, Nanjing, China;

**PSWC**Philip S. Ward collection, University of California, Davis, CA, USA;

**UCDC**Bohart Museum of Entomology, University of California, Davis, CA, USA.

## Results

### Hymenoptera Linnaeus, 1758

#### Apocrita Latreille, 1810

**Aculeata Latreille, 1802**

##### Formicoidea

Taxon classificationAnimaliaHymenopteraFormicidae

Latreille, 1809

ED4248E9-AD77-5762-AFDC-2F714D780049

###### Definition.

Detailed study of the †*Camelomecia* clade has redefined the Formicoidea and refined our understanding of the definition and evolutionary patterning of the total and crown Formicidae (Boudinot et al. 2020a). Formicoids, we now know, are a clade of Formicapoidina (sister to Apoidea: Johnson et al. 2013; Branstetter et al. 2017; Peters et al. 2017) defined by positive (i.e., non-“absence” character) morphological synapomorphies most of which form an innovation suite for cursorial or surface-based predation, including: **(1)** prognathy and elongation of the postgenal bridge (Figs 13A, 14B); **(2)** enlargement of the dorsal (cranial) mandibular condyle (Fig. 13A); **(3)** rotation of the antennal toruli laterad in females (Fig. 13A, B); **(4)** elongation of the procoxae (Figs 14A, 15A); **(5)** partial to complete enclosure of the proximal protrochanteral articulations within the distal procoxal foramina (Figs 14E, 15A, 16E); **(6)** internalization of the proximal meso- and metacoxal articulations within the mesosoma (Figs 14B, 15A, 16); **(7)** petiolation of the first metasomal segment (Figs 14A, 15A, 16A, D, E); **(8)** gain of the anteroventral process of the petiolar sternum (Fig. 16A); **(9)** buttressing of the metasomal waist through gain of the prora (an anteroventral process of the second metasomal sternum) (Figs 15A, 16A, D); plus **(10)** an angled juncture between the first free abscissae of Rs and M in the fore wing (Figs 15A, 16A, D). The †*Camelomecia* clade, in contrast to the total clade of the Formicidae, probably lack the metapleural gland and apterous workers altogether, while also being defined by a combination of derived and plesiomorphic features (see, e.g., the key below). Based on direct examination of the unique specimen (holotype) of †*Camelomecia janovitzi* (BEB at the AMNH, 2017), presence of this gland is uncertain and requires further scrutiny.

### Extended definition of diagnostic states

To ensure maximal clarity for the definition provided for the Formicoidea above, we provide further definition of these conditions here:

**(1)** Prognathy in ants is achieved through elongation of the postgenal bridge, i.e., the sclerotization between the occipital and oral foramina (Richter et al. 2019, 2020). This condition has arisen independently in a number of other Aculeata, including the Bethylidae, Dryinidae, Sclerogibbidae, a few Tiphioidea, some Mutillidae, Philanthidae, and Ampulicidae. This was recognized as a synapomorphy of the Formicidae by Bolton (2003).

**(2)** The dorsal mandibular condyle–also known as the “cranial condyle” or “anterior mandibular condyle” in hypognathous taxa (Snodgrass 1935, 1956) and more recently labeled the “dorsal mandibular articulation” (Richter et al. 2019, 2020, Klunk et al. 2020)–is an outgrowth of the cranium which articulates with the mandible via the mandibular acetabulum, forming the second functional condyle defining the Dicondylia. In ants, this condyle is much enlarged, allowing for a wider gape via a slide-locking mechanism. The form is variable among and often within the crown ant subfamilies, and the enlarged condition is a strong diagnostic feature of the Formicoidea, with limited similarity among other Aculeata.

**(3)** Torular orientation is mechanically significant and has consequences for the radicle form. The ancestral condition of the Aculeata is to have the toruli directed anteriorly away from the cranium (assuming hypognathy), such that the foramina are aligned with the plane of the cranium, nearly perpendicular to the long axis of the head. In the majority of Formicoidea, the medial rims of the toruli are raised dorsally (assuming prognathy), such that they are above the lateral rims when viewed in profile; when viewed anteriorly, the foramina of the toruli are clearly directed laterally. This condition has arisen in a number of other Aculeata, so the diagnostic value of this is limited to that of the cranial condyle.

**(4)** Procoxal elongation is observed in ants when compared to other Aculeata, as recognized by Liu et al. (2019). Specifically, the procoxae are ca. 2 × as long dorsoventrally as they are wide in anteroposterior diameter. This is another strong diagnostic feature as the procoxae of most other Aculeata are generally more globular or are somewhat wedge-shaped in profile view, with lengths sometimes only slightly exceeding widths, even in lineages with apterous females.

**(5)** Distal protrochanteral closure is the condition in which the coxotrochanteral articulation is concealed within the procoxa, such that the membrane is completely hidden (Boudinot 2015). This is observed in all living ants, including males and deformed inquilines, and was confirmed for all amber taxa for which the condition could be evaluated, including stem Formicidae plus the †*Camelomecia* group. In a number of fossils examined, the articulation of the procoxa and trochanter is decayed, giving the appearance of a partially open cavity. In these cases, the strong constriction and curvature of the protrochanter are observable and indicative of a tighter articulation prior to death. Among all Aculeata, protrochanteral closure is only observed in some species of *Myrmosa* (Mutillidae), but these insects are otherwise grossly distinct from Formicoidea.

**(6)** Closure of the meso- and metathoracicocoxal articulations is the condition in which the proximal articulatory structures of the coxae are completely internalized within the mesosoma, resulting in total concealment of the articulatory membranes. This is distinct from closure of the metacoxal cavity by a ring of sclerite, which relates to the separation or lack thereof from the propodeal foramen (Bolton 2003, Keller 2011). The proximal coxal articulations are further modified as ball-like structures, while the thoracic foramina are oriented parallel to the ground (Boudinot 2015, Liu et al. 2019). Closure of these thoracicocoxal articulations is much more frequently observed among Aculeata than closure of the distal procoxal articulation and is widespread among those taxa with apterous females.

**(7)** Petiolation of the first metasomal segment is the condition in which the posterior foramen is constricted, and in ants is associated with the formation of a distinct posterior face of the muscular dorsal node. This was recognized as a synapomorphy of the Formicidae by Bolton (2003). It should be noted that petiolation of the second metasomal segment is observed as a spectrum, with varying degrees of constriction and reduction in size.

**(8)** The subpetiolar process is a cuticular projection of the petiolar sternum which articulates between the metacoxae when the metasoma is completely down-flexed. The process is present in the majority of the Formicoidea, and similar structures have been gained in some non-ant Aculeata, including a few Chrysididae, a few Tiphioidea, some Ampulicidae, some Bembicidae, and some Philanthidae. The subpetiolar process was intuitively inferred to be absent in the ancestral formicid by Bolton (2003, p. 289), whereas it is robustly supported a synapomorphy of the superfamily in our work.

**(9)** The prora is an anteroventral thickening of the second metasomal sternum that buttresses the segment during strong ventral flexion of the abdomen. Presence of the prora has not been previously recognized as a defining feature of the ants, and similar developments are observed in only a few other Aculeata, such as Brachycistidinae (Tiphiidae), *Chyphotes* (Chyphotidae), and *Dolichurus* (Ampulicidae). Loss of the prora has occurred sporadically among the crown Formicidae, and defines, for example, the Formicinae as well as the Aneuretinae + Dolichoderinae clade.

**(10)** An angled juncture of Rsf1 and Mf1 is observed with some frequency among other Aculeata and has been reversed or otherwise modified in various crown Formicidae. Among venational features, Bolton (2003) recognized loss of 3rs-m and 2m-cu as synapomorphies of the Formicidae; these are observed to be present in some but not all †*Camelomecia* group species, indicating tendency for parallel loss.

#### Synopsis of Formicoidea emphasizing Mesozoic taxa

**Note.** Only taxa known from the Mesozoic are listed. For a complete subfamily-level classification of crown Formicidae as stands, see AntCat.org. †Armaniinae Dlussky, 1983 and other compression-fossil taxa are treated in a forthcoming phylogenetic study, as will the Burmite fossil †*Burmomyrma rossi* Dlussky, 1996, which was recently transferred from the Formicidae to the †Falsiformicidae by Lucena and Melo (2018) without morphological justification. Note that the revised diagnosis of †*Myanmyrma* is provided in the key, particularly couplet 10. Elevation of the †Zigrasimeciinae stat. nov. is justified by their morphological distinctness from all other Formicidae and uncertain relationship with other stem groups (see the key and Cao et al. 2020). Moreover, this action stabilizes the formalization of stem subfamilies, following the similar elevation of †Haidomyrmecinae by Perrichot et al. (2020). Given the expanding knowledge of haidomyrmecines, recognition of a tribal system may be worthwhile. Regarding the poorly preserved fossils attributed to †*Cretomyrma*, we have made this transfer based on the results of Boudinot et al. (2020a); for which a refined explanation will be provided soon. Bracketed abbreviations in the list below indicate sex or caste: f = female/gyne, m = male, w = worker.

†*Camelomecia* clade

1• †*Camelomecia* Barden & Grimaldi, 2016

1. †*Cm. janovitzi* Barden & Grimaldi, 2016 [f, Burmese amber]

2• †*Camelosphecia* gen. nov.

2. †*Cs. fossor* sp. nov. [f, Burmese amber]

3. †*Cs. venator* sp. nov. [m, Burmese amber]

Total clade Formicidae Latreille, 1809

Stem Formicidae*incertae sedis*

3• †*Baikuris* Dlussky, 1987

4. †*Ba. casei*Grimaldi et al., 1997 [m, Raritan amber]

5. †*Ba. mandibularis* Dlussky, 1987 [m, Taimyr amber]

6. †*Ba. maximus* Perrichot, 2015 [m, Charentese amber]

7. †*Ba. mirabilis* Dlussky, 1987 [m, Taimyr amber]

4• †*Cretomyrma* Dlussky, 1975 subfam. transfer

8. †*Cr. arnoldii* Dlussky, 1975 [w, Taimyr amber]

9. †*Cr. unicornis* Dlussky, 1975 [w, Taimyr amber]

5• †*Dlusskyidris* Bolton, 1994

10. †*Dl. zherichini* Bolton, 1994 [m, Taimyr amber]

Clade †Sphecomyrmines nom. nov.

†Haidomyrmecinae Bolton, 2003 (see Barden et al. 2020; Perrichot et al. 2020)

†*Aquilomyrmex* clade

6• †*Aquilomyrmex*Perrichot et al., 2020

11. †*A. huangi*Perrichot et al., 2020 [f, Burmese amber]

7• †*Chonidris*Perrichot et al., 2020

12. †*Ch. insolita*Perrichot et al., 2020 [f, Burmese amber]

8• †*Dhagnathos*Perrichot et al., 2020

13. †*Dh. autokrator*Perrichot et al., 2020 [f, Burmese amber]

†*Haidomyrmex* group (newly recognized)

9• †*Dilobops* Lattke & Melo, 2020

14. †*Di. bidentata* Lattke & Melo, 2020 [w, Burmese amber]

†*Haidomyrmex* clade

†*Ceratomyrmex* subclade

10• †*Ceratomyrmex*Perrichot et al., 2016

15. †*Ce. ellenbergeri*Perrichot et al., 2016 [w, Burmese amber]

16. †*Ce. planus* Lattke & Melo, 2020 [w, Burmese amber]

11• †*Linguamyrmex* Barden & Grimaldi, 2017

17. †*L. brevicornis*Perrichot et al., 2020 [f, w, Burmese amber]

18. †*L. rhinocerus* Miao & Wang, 2019 [w, Burmese amber]

19. †*L. vladi* Barden & Grimaldi, 2017 [w, Burmese amber]

12• †*Protoceratomyrmex*Perrichot et al., 2020

20. †*Pc. revelatus*Perrichot et al., 2020 [w, Burmese amber]

†*Haidomyrmex* subclade

13• †*Haidomyrmex* Dlussky, 1996

21. †*Hx. cerberus* Dlussky, 1996 [w, Burmese amber]

22. †*Hx. davidbowiei* Lattke & Melo, 2020 [w, Burmese amber]

23. †*Hx. scimitarus* Barden & Grimaldi, 2012 [f, Burmese amber]

24. †*Hx. zigrasi* Barden & Grimaldi, 2012 [f, Burmese amber]

14• †*Haidomyrmodes*Perrichot et al., 2008

25. †*Hd. mammuthus*Perrichot et al., 2008 [f, w, Charentese amber]

15• †*Haidoterminus*McKellar et al., 2013b

26. †*Ht. cippus*McKellar et al., 2013b [w, Medicine Hat amber]

†Zigrasimeciinae Borysenko, 2017 stat. nov. (see also Cao et al. 2020)

16• †*Boltonimecia* Borysenko, 2017

27. †*Bo. canadensis* (Wilson, 1985) [w, Medicine Hat amber]

17• †*Protozigrasimecia*Cao et al., 2020

28. †*Pz. chauli*Cao et al., 2020 [w, Burmese amber]

18• †*Zigrasimecia* Barden & Grimaldi, 2013 (gynes known, see also Cao et al. 2020)

29. †*Z. ferox* Perrichot, 2014 [w, Burmese amber]

30. †*Z. hoelldobleri*Cao et al., 2020 [w, Burmese amber]

31. †*Z. tonsora* Barden & Grimaldi, 2013 [w, Burmese amber]

†Sphecomyrminae Wilson & Brown, 1967 *sensu stricto*

19• †*Gerontoformica* Nel & Perrault, 2004 (see Barden and Grimaldi 2014)

†*Gerontoformica orientalis* species group (newly recognized; †*Gerontoformicasensu stricto*)

32. †*G. cretacica* Nel & Perrault, 2004 [w, Charentese amber]

33. †*G. gracilis* (Barden & Grimaldi, 2014) [w, Burmese amber]

34. †*G. occidentalis* (Perrichot et al., 2008) [w, Charentese amber]

35. †*G. orientalis* (Engel & Grimaldi, 2005) [w, Burmese amber]

• (Note: †*G. orientalis* is the type species of †*Sphecomyrmodes* Engel & Grimaldi, 2005)

36. †*G. robusta* (Barden & Grimaldi, 2014) [w, Burmese amber]

37. †*G. spiralis* (Barden & Grimaldi, 2014) [w, Burmese amber]

38. †*G. subcuspis* (Barden & Grimaldi, 2014) [w, Burmese amber]

†*Gerontoformica pilosa* species group (newly recognized)

39. †*G. contega* (Barden & Grimaldi, 2014) [w, Burmese amber]

40. †*G. magna* (Barden & Grimaldi, 2014) [w, Burmese amber]

41. †*G. pilosa* (Barden & Grimaldi, 2014) [w, Burmese amber]

†*Gerontoformica* species newly recognized as unplaceable due to preservation

42. †*G. rugosa* (Barden & Grimaldi, 2014) [w, Burmese amber]

43. †*G. tendir* (Barden & Grimaldi, 2014) [w, Burmese amber]

20• †*Myanmyrma* Engel & Grimaldi, 2005

• (Note: The diagnosis of †*Myanmyrma* is revised via the key below, see particularly couplet 10)

44. †*M. gracilis* Engel & Grimaldi, 2005 [w, Burmese amber]

45. †*M. maraudera* (Barden & Grimaldi, 2014) comb. nov. [w, Burmese amber]

21• †*Sphecomyrma* Wilson & Brown, 1967

46. †*S. freyi* Wilson & Brown, 1967 [w, Raritan amber]

47. †*S. mesaki* Engel & Grimaldi, 2005 [w, Raritan amber]

Clade Antennoclypeata nom. nov.

†Brownimeciinae Bolton, 2003

22• †*Brownimecia*Grimaldi et al., 1997

48. †*Br. clavata*Grimaldi et al., 1997 [w, Raritan amber]

Crown clade Formicidae Latreille, 1809

Ponerinae Lepeletier de Saint-Fargeau, 1835

• Kachin and Tilin burmite deposits (Zheng et al. 2018, unpubl. data).

Dolichoderinae Forel, 1878

23• †*Chronomyrmex*McKellar et al., 2013a

49. †*Cx. medicinehatensis*McKellar et al., 2013a [w, Medicine Hat amber]

• Kachin and Tilin burmite deposits (Zheng et al. 2018, unpubl. data).

Formicinae Latreille, 1809

24• †*Kyromyrma* Grimaldi & Agosti, 2000

50. †*K. neffi* Grimaldi & Agosti, 2000 [w, Raritan amber]

• Kachin and Tilin burmite deposits (Zheng et al. 2018, unpubl. data).

Additional crown taxa:

25• †*Canapone* Dlussky, 1999 (“Ponerinae” *sensu* Wilson, Brown, and others)

51. †*Cp. dentata* Dlussky, 1999 [w, Medicine Hat amber]

26• †*Cananeuretus* Engel & Grimaldi, 2005 (Aneuretinae Emery, 1913)

52. †*Cn. occidentalis* Engel & Grimaldi, 2005 [w, Medicine Hat amber]

### Diagnostic key to the major groupings of Mesozoic Formicoidea

**Note.** †Armaniinae and other compression fossil taxa are not included in this key due to a lack of preserved detail. Additionally, †*Burmomyrma* and †*Cretomyrma* are excluded from this key as both fossils are missing their anterior halves. Comprehensive review of all Mesozoic male Formicoidea is necessary before †*Baikuris* and †*Dlusskyidris* can be considered specifically identifiable. For detailed keys to the genera and species of †Haidomyrmecinae, we refer the readers to Perrichot et al. (2020); in distinction to the former publication, we include †*Dilobops* which was published soon thereafter (Lattke and Melo 2020).

**Table:** 

1	Mandibles of both sexes cup-shaped, being strongly bowed in lateral view (Figs 1A, 2). Masticatory margins of mandibles in both sexes always elongate (white triangle, Fig. 1A). Entire body of clypeus projecting anteriorly away from remainder of cranium (black triangle, Fig. 1A) (weakly so in males). Anterior clypeal margin always lacking chaetae (“traction setae”, “spicules”, “pegs”). Frontal carinae (paired median, longitudinal ridges) between antennal toruli always absent. Face always without anterior or dorsomedian projections	**2 († *Camelomecia* clade)**
–	Mandibles not cup-shaped (Fig. 1B, C). Masticatory margins of mandibles short (white triangle, Fig. 1B) or elongate (white triangle, Fig. 1C). Body of clypeus not projecting anteriorly away from remainder of cranium, rather being tightly integrated and surrounded laterally by the malar area. Anterior clypeal margin with or without chaetae (arrows, Fig. 1B, C). Frontal carinae usually present	**3 (Formicidae Latreille, 1809)**
2	Masticatory mandibular margins of both sexes multidentate, with ten or more well-defined teeth (Fig. 2A). Anterior clypeal margin of female produced anteriorly as thin, laminar sheet (black triangle, Fig. 1A). Female anterior clypeal margin medially bidentate (white curve and arrows, Fig. 2A). Disc (i.e., dorsal surface) of female labrum with several very large, stout, and curved chaetae (black asterisks, Fig. 2A). Compound eyes of female massive, taking up most of cranium laterally. Female profemora massively enlarged. Second protarsomere of female margined with conspicuous psammochaetae (“fossorial setae”)	† ***Camelosphecia* gen. nov.**
–	Masticatory mandibular margins of both sexes edentate, or if teeth present, these very fine, representing mere crenulation (Fig. 2B). Anterior clypeal margin not produced anteriorly as thin laminar process. Anterior clypeal margin edentate, but may be medially emarginate (white curve, Fig. 2B). Disc of female labrum glabrous, lateral margins with sprays of thin setae (white asterisks, Fig. 2B). Female compound eyes comparatively small, not taking up entire lateral side of cranium. Female profemora thin, twig-like. Second protarsomere of female lacking psammochaetae	† ***Camelomecia* Barden & Grimaldi, 2016**
3	Basal angle or tooth of mandible (i.e., the juncture between the basal and masticatory margins) directed posterodorsally (black curve on mandible, Fig. 3A), whether as an elongate process or simply due to rotation of the mandibles. Clypeus or anterior region of face distinctly produced, either bearing a tubercle or laminar or linear processes (white curve on clypeus, Fig. 3A). Elongate (trigger) setae present or absent on face projecting into active area of mandibles (black arrows, Fig. 3A). (Note: Males unknown.)	**4 (†Haidomyrmecinae Bolton, 2003**; see Barden et al. 2020 for phylogeny)
–	Basal angle or tooth of mandible never directed posterodorsally (black curve on mandible, Fig. 3B). Clypeus or anterior head region not produced, nor bearing such processes (white curve on clypeus, Fig. 3B). Elongate setae absent. (Note: Males known.)	**7**
4	Labrum large and exposed beneath clypeus (white curves, Fig. 4A, B) (ventral view or open mandibles sometimes necessary, as in Fig. 4B). Labrum with peg-like chaetae (natural black structures outside of white curve in Fig. 4A, inside curve in Fig. 4B). Median portion of clypeus ecarinate, i.e., lacking median longitudinal carina. Clypeus and labrum both concave in cross-section. Trigger hairs absent	†***Aquilomyrmex* clade** (see couplet 9 of Perrichot et al. 2020 to distinguish among constituent genera)
–	Labrum small and concealed in buccal cavity. Labrum without margin or field of peg-like chaetae. Median portion of clypeus with or without a median longitudinal carina. Clypeus and labrum not concave; clypeus flat to convex in cross-section (black curve beneath frontal process, Fig. 4C). Trigger hairs present as long, thin setae which project into the active area of the mouthparts (black arrow, Fig. 4C)	**5 (†*Haidomyrmex* group)**
5	Clypeus with a dense brush of chaetae (brush indicated by white asterisk, Fig. 5A); brush located approximately in center of clypeus or more dorsally, just beneath the frontal tubercle	**6**
–	Clypeus without a dense brush of chaetae; frontal horn, if present, may bear long, thin setae or be apically margined by short, peg-like chaetae (short thick hairs outside of white curve in Fig. 5B)	†***Ceratomyrmex* subclade** (see couplet 6 of Perrichot et al. 2020 to distinguish among constituent genera)
6	Cranium highly modified: In profile view, cranium more-or-less teardrop-shaped (Fig. 6A); clypeal area dorsoventrally elongate, concave (black curve, Fig. 6A); mouthparts migrated posteroventrally, thus nearly in a hypognathous position. Anterolateral corners of cranium simple, without distinct triangular processes	†***Haidomyrmex* subclade** (see couplet 2 of Perrichot et al. 2020 to distinguish genera of this group)
–	Cranium not modified as above: cranium not teardrop-shaped; clypeal area not dorsoventrally elongate; and mouthparts not migrated posteroventrally. Anterolateral corners of cranium armed with distinct triangular processes (black angle, Fig. 6B)	† ***Dilobops* Lattke & Melo, 2020**
7	Clypeus extending posteriorly past the anterior margins of the antennal toruli (right torulus on left side, as shown, marked by white curve, Fig. 7A), thus having an elongate posteromedian strip between the antennal insertions (black curve, Fig. 7A); exceptions within the crown clade include Pseudomyrmecinae and many Formicinae (see Bolton 1994, 2003; Boudinot 2015 for identification). Anterolateral portions of clypeus **not** developed as lobate processes; anterolateral clypeal corners or malar area may be dentate. Mandibles edentate to many-dentate. Scape length > 4 × scape width and ≥ 1/2 × head length	**8 (Antennoclypeata)**
–	Clypeus not extending posteriorly past anterior margins of the antennal toruli (right torulus on left side, as shown, marked by white curve, Fig. 7B), thus without an elongate posteromedian strip between the antennal insertions (black curve, Fig. 7B). Anterolateral portions of clypeus developed as broad and flat lobe-like processes overhanging or tightly fitting against mandibular bases (white arrows pointing at white curve, Fig. 7B). Mandibles uni- or bidentate (Fig. 7B). Scape length < 4 × scape width and ≤ 1/2 × head length	**9**
8	Mandible unidentate. Cranium dome-shaped (deep black curve at back of head, Fig. 8A). Anterolateral corners of cranium (malar area) derived as pointed, triangular processes (black angle near front of head, Fig. 8A). Anterolateral margins of mesopectus evenly rounded, without longitudinal (epicnemial) carina. (Queens and males unknown.)	†**Brownimeciinae Bolton, 2003**
–	Mandibles uni- or more dentate. Cranium not dome-shaped (shallow black curve at back of head, Fig. 8B). Anterolateral corners of cranium not (black curve near mandibles, Fig. 8B) or rarely derived as pointed, triangular processes (Amblyoponinae Forel, 1893). Anterolateral margins of mesopectus often angularly marked by longitudinal (epicnemial) carina (white curve, Fig. 8B). (Note: Queens and males known)	**crown clade Formicidae**
9	Clypeus transverse and arcuate; anterior clypeal margin broadly concave (Fig. 9B). Mandible rotated in socket with blade torqued such that the ventromedial mandibular margin is exposed in full-face view (black line exposed proximal to white curve of basal margin, Fig. 9A) (state unknown for †*Boltonimecia*). Ventral (inner) mandibular face with dense array of spiniform chaetae (state unknown for †*Boltonimecia*). Antennal toruli very wideset, being nearly situated beneath compound eyes in full-face view, approximately one scape length or more apart. Facial region of cranium in female castes with linear and diagonally oriented scrobes for reception of antennal scapes, extending from antennal toruli to anterior margins of compound eyes. (Note: Males unknown.)	†**Zigrasimeciinae Borysenko, 2017, stat. nov.** (see Cao et al. 2020 for key to constituent genera)
–	Clypeus variable, usually shield like; anterior clypeal margin linear to convex (Fig. 9C) or medially emarginate. Mandible neither rotated in socket nor blade torqued; ventromedial margin of mandible only visible laterally (Fig. 9C). Antennal toruli close-set, positioned well medial to the compound eyes in full-face view, and distinctly less than one scape length apart. Facial region of cranium in female castes without diagonally oriented scrobes; if areas lateral to frontal carinae sulcate, these sulci are curved and ending medial to compound eye or posterior to compound eye anterior margin (†*Myanmyrma* is the exception, Fig. 10A). (Note: Males known.)	**10 (†Sphecomyrminae Wilson & Brown, 1967 sensu stricto**, including male-based taxa †Baikuris Dlussky, 1987 and †Dlusskyidris Bolton, 1994 [see also: Grimaldi et al. 1997, Perrichot 2015]; for putative male of †Sphecomyrma Wilson & Brown, 1967, see Grimaldi et al. 1997)
10	Mandibles elongate, not fitting against clypeus snugly at rest. Frontal carinae robustly flanged, only weakly curved along their lengths, and ending posterior to anterior eye margin (white curve, Fig. 10A) (state not exactly known for †*M. gracilis*). Both clypeus and labrum bearing chaetae on their contact surfaces. (Note: Males unknown)	† ***Myanmyrma* Engel & Grimaldi, 2005**
–	Mandibles short, fitting snugly against clypeus at rest. Frontal carinae finely carinate, strongly curved thus forming a distinct semicircle, and ending anterior the anterior eye margin (white curve, Fig. 10B). Rarely both clypeus and labrum with chaetae on their contact surfaces. (Note: Males known)	**11**
11	Clypeus lacking chaetae. Clypeus bearing lateromedially narrow anteromedian lobate process which overhangs mandibles at mandibular closure (white curve, Fig. 11A). Abdominal segment III foreshortened	† ***Sphecomyrma* Wilson & Brown, 1967**
–	Clypeus with chaetae. Clypeus more-or-less evenly convex, without a distinct anteromedian lobate process (white curve, Fig. 11B). Abdominal segment III foreshortened or not	**12 (†Gerontoformica Nel & Perrault, 2004**; see Barden and Grimaldi 2014 for key to species)
12	Abdominal segment IV with tergum and sternum strongly differentiated into pre- and post-sclerites by sulci and a deep constriction ("cinctus") (Fig. 12A). Abdominal segment III not foreshortened; its anteroposterior length usually greater than that of petiole (Fig. 12A)	† ***Gerontoformica pilosa* species group**
–	Abdominal segment IV with tergum and sternum weakly or not at all differentiated into pre- and post-sclerites by sulci or a constriction (Fig. 12B). Abdominal segment III usually anteroposteriorly foreshortened; its anteroposterior length usually less than that of petiole (Fig. 12B)	† ***Gerontoformicasensu stricto***

**Figure 1.**
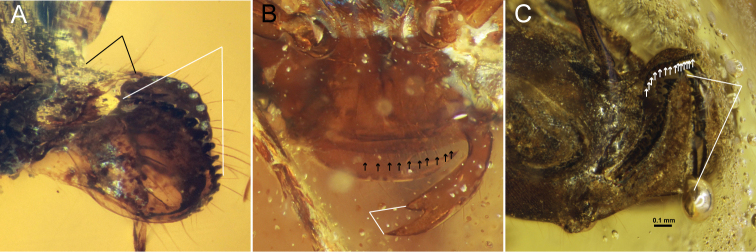
Mandibles of representative Formicoidea**A** †*Camelosphecia fossor* gen. et sp. nov. holotype female, lateral oblique (ANTWEB1038930) **B** †*Gerontoformica* species, male, dorsal or full-face view (ANTWEB1032638) **C** †*Chonidris insolita* holotype female, anterolateral oblique (FANTWEB00022, AntWeb: Vincent Perrichot).

**Figure 2.**
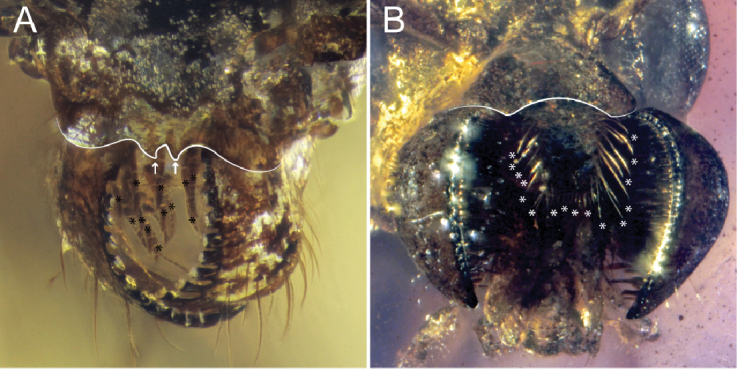
Mouthparts of †*Camelomecia* clade genera **A** †*Camelosphecia fossor* gen. et sp. nov. holotype female, dorsolateral oblique (ANTWEB1038930) **B** †*Camelomecia janovitzi* holotype female, anterolateral oblique (AMNH-BUTJ003, AntWeb: Phil Barden).

**Figure 3.**
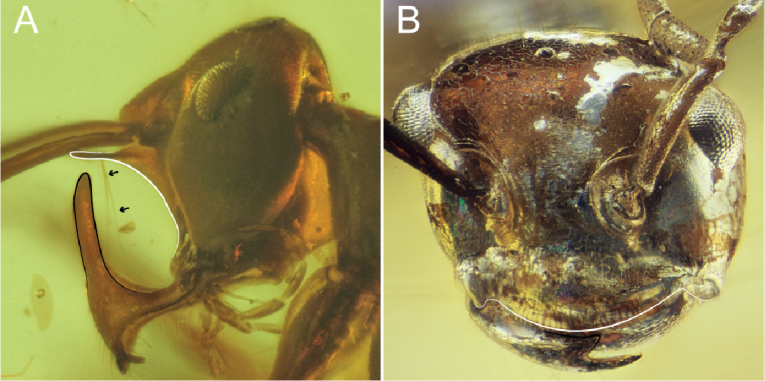
Cranium and mandibles of stem Formicidae**A** †*Linguamyrmex brevicornis*, worker, lateral view (FANTWEB00035, AntWeb: Vincent Perrichot) **B** †*Gerontoformica* species, worker, dorsolateral oblique (ANTWEB1032639).

**Figure 4.**
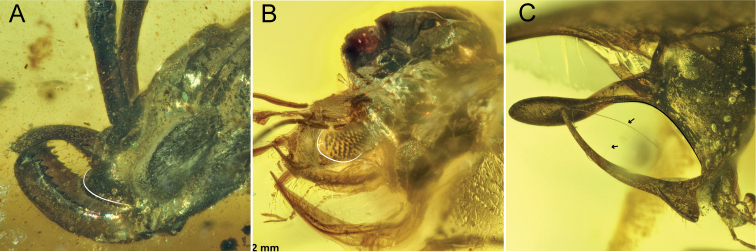
Cranial armaments of †Haidomyrmecinae**A** †*Chonidris insolita* female, dorsolateral oblique (FANTWEB00033, AntWeb: Vincent Perrichot) **B** †*Aquilomyrmex huangi* female, ventrolateral oblique (FANTWEB00023, AntWeb: Vincent Perrichot) **C** †*Linguamyrmex rhinocerus*, female, lateral (FANTWEB00016, AntWeb: Vincent Perrichot).

**Figure 5.**
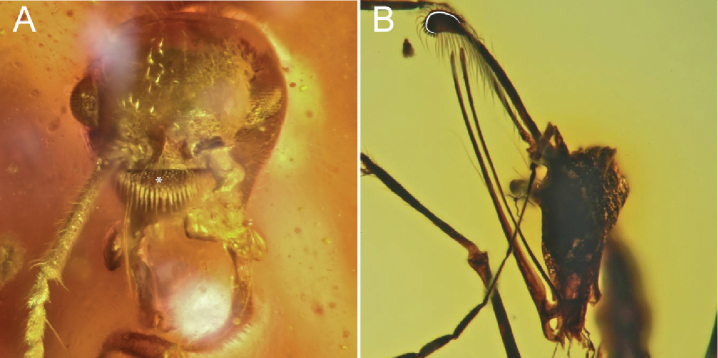
Facial seta and chaeta arrays of trigger-hair-bearing †Haidomyrmecinae**A** †*Haidomyrmex cerberus*, holotype worker, dorsolateral oblique (BMNHP20182, AntWeb: Vincent Perrichot) **B** †*Ceratomyrmex ellenbergeri*, worker, dorsal anterolateral oblique (FANTWEB00005, AntWeb: Vincent Perrichot).

**Figure 6.**
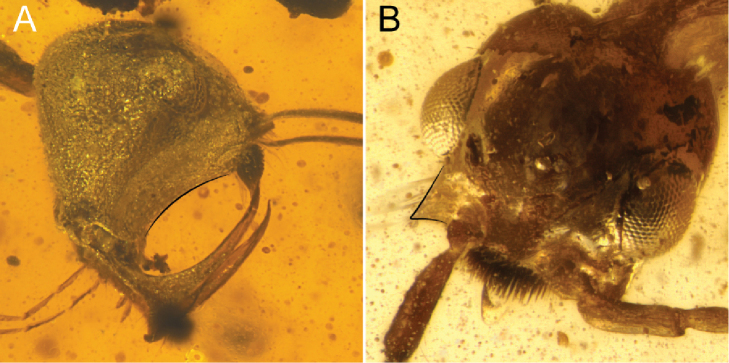
Cranial conformation of clypeal-brush-bearing †Haidomyrmecinae**A** †*Haidomyrmex cerberus* worker, lateral (FANTWEB00017, AntWeb: Vincent Perrichot) **B** †*Dilobops bidentata* worker holotype, dorsolateral oblique (FANTWEB00039, AntWeb: Gabriel Melo).

**Figure 7.**
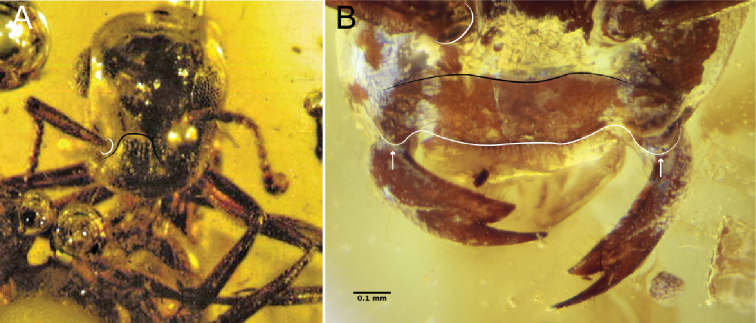
Clypeus and cranium of Antennoclypeata and †Sphecomyrmines **A** †*Brownimecia clavata* holotype worker, full-face (from Grimaldi and Engel 2005, used with permission of the publisher) **B** †*Gerontoformica* species, worker, dorsal (ANTWEB1032629).

**Figure 8.**
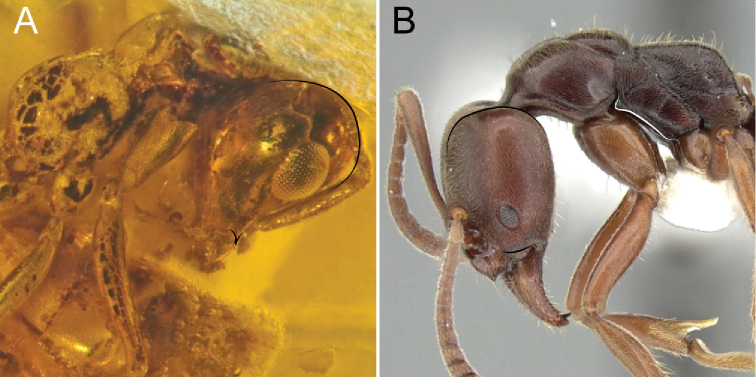
Cranial and mesosomal profiles of Antennoclypeata **A** †*Brownimecia clavata* holotype worker, lateral (AMNH-NJ667, AntWeb: Dave Grimaldi and Vincent Perrichot) **B***Austroponera castanea* (Mayr, 1865) worker, lateral (CASENT0249168, AntWeb: Ryan Perry).

**Figure 9.**
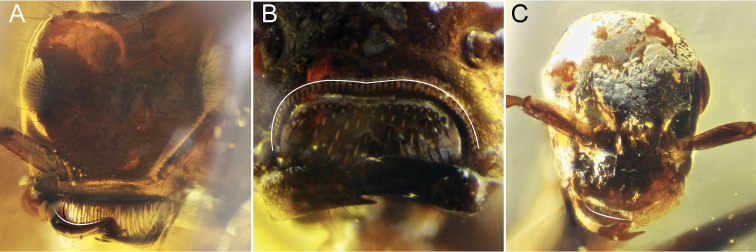
Facial views of †Zigrasimeciinae stat. nov. and †Sphecomyrminae*sensu stricto***A** †*Zigrasimecia* species, worker, posterodorsal oblique (ANTWEB1032623) **B** †*Zigrasimecia* species, worker, anterodorsal oblique (ANTWEB1032660) **C** †*Gerontoformica* species, worker, dorsal (ANTWEB1032649).

**Figure 10.**
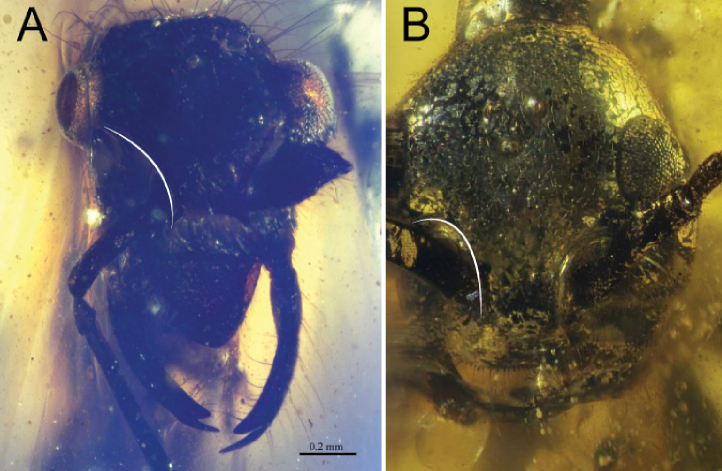
Cranial gestalts of †Sphecomyrminae*sensu stricto***A** †*Myanmyrma maraudera* (Barden & Grimaldi, 2016) comb. nov., holotype worker, full-face (JZCBU1846, AntWeb: Phillip Barden) **B** †*Gerontoformica* species, worker, full-face (ANTWEB1038348).

**Figure 11.**
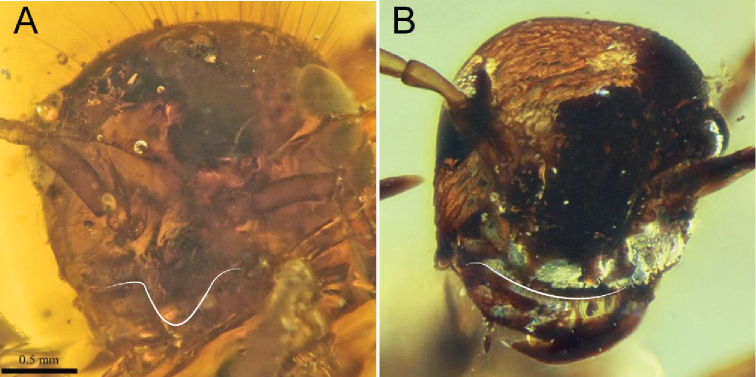
Facial views of †Sphecomyrminae which have short mandibles **A** †*Sphecomyrma mesaki* Engel & Grimaldi, 2005, holotype worker, anterodorsal oblique (AMNH-NJ1023, AntWeb: Dave Grimaldi and Vincent Perrichot) **B** †*Gerontoformica* species, worker, full-face (ANTWEB1032418).

**Figure 12.**
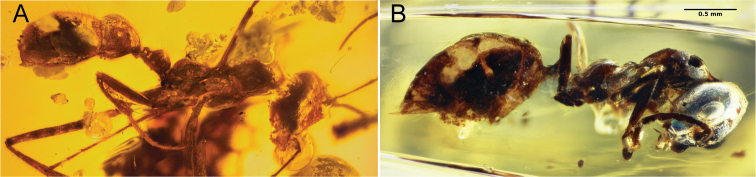
Body profiles of †*Gerontoformica pilosa* and *orientalis* species groups **A** †*Gerontoformica pilosa* Barden & Grimaldi, 2014, worker profile (ANTWEB1038931) **B** †*Gerontoformica* species, worker profile (ANTWEB1032649).

### New taxon definitions

#### † Camelosphecia gen. nov.

Taxon classificationAnimaliaHymenopteraFormicidae

748D3BAB-577E-5A1C-8485-08F1BC686DC9

http://zoobank.org/5E38E92B-51D4-4B0B-B8DA-FAE77F7764B9

**Figure 13.**
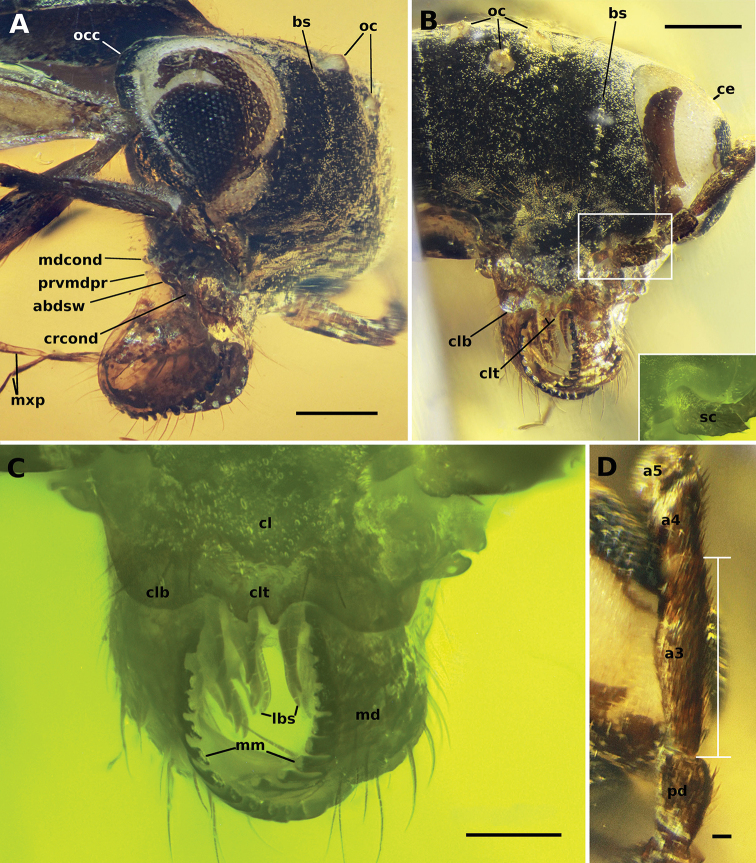
Holotype of †*Camelosphecia fossor* sp. nov. **A** lateral view of right side of head **B** full face view of head, right margin blurred on this view due to a folding of the facet. Box on bottom right evidencing scape under fluorescent microscopy **C** detail of clypeus, mandibles and labrum under fluorescent microscopy **D** posterodorsal view of anterior section of funiculus (pedicel plus flagellum), evincing pedicel and antennomeres III and IV. Abbreviations: **a3–5**, antennomeres III–V; **abdsw**, abductor swelling; **bs**, bubble stream; **ce**, compound eye; **cl**, clypeus; **clb**, clypeal lobe; **clt**, clypeal teeth; **crcond**, cranial condyle; **md**, mandible; **mm**, masticatory margins; **lbs**, labral chaetae; **mdcond**, mandible condyle; **mxp**, maxillary palps; **oc**, ocelli; **occ**, occipital carina; **pd**, pedicel; **prvmdpr**, posteroventral mandible process; **sc**, scape. Scale bars: 0.2 mm (**A, B**); 0.1 mm (**C**); 0.02 mm (**D**).

**Figure 14.**
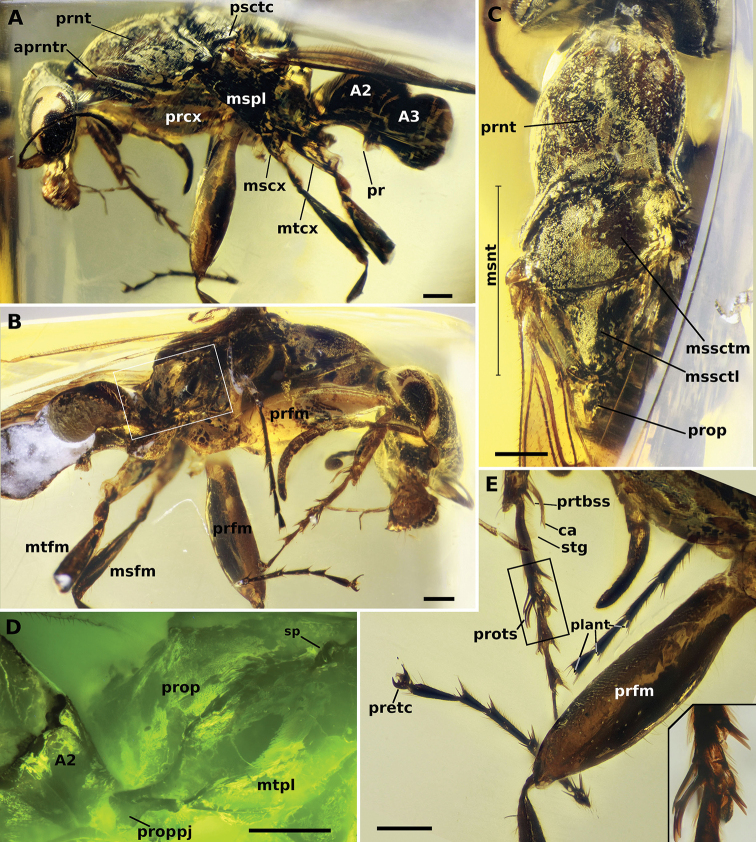
Holotype of †*Camelosphecia fossor* sp. nov. **A** profile of left side of body **B** profile of right side of body **C** dorsal view of mesosoma **D** delimited area of **C** under fluorescent light **E** details of the legs, especially the forelegs. Abbreviations: **A2–A3**; abdominal segments 2 and 3; **aprntr**, anterior pronotal rim; **ca**, calcar; **mscx**, mesocoxa; **msfm**, mesofemur; **msnt**, mesonotum; **mspl**, mesopleural region of mesopectus; **mssctm**, mesoscutum; **mssctl**, mesoscutellum; **mtcx**, metacoxa; **mtfm**, metafemur; **mtpl**, metapleural region of metapectus; **plant**, plantulae; **pr**, prora; **prcx**, procoxa; **pretc**, pretarsal claws; **prfm**, profemur; **prnt**, pronotum; **prop**, propodeum; **proppj**, propodeal projection; **prots**, protarsomeres I and II chaetae; **prtbss**, distal protibia stout chaeta; **psctc**, parascutal carina; **sp**, propodeal spiracle; **strg**, strigil. Scale bars: 0.2 mm.

**Figure 15.**
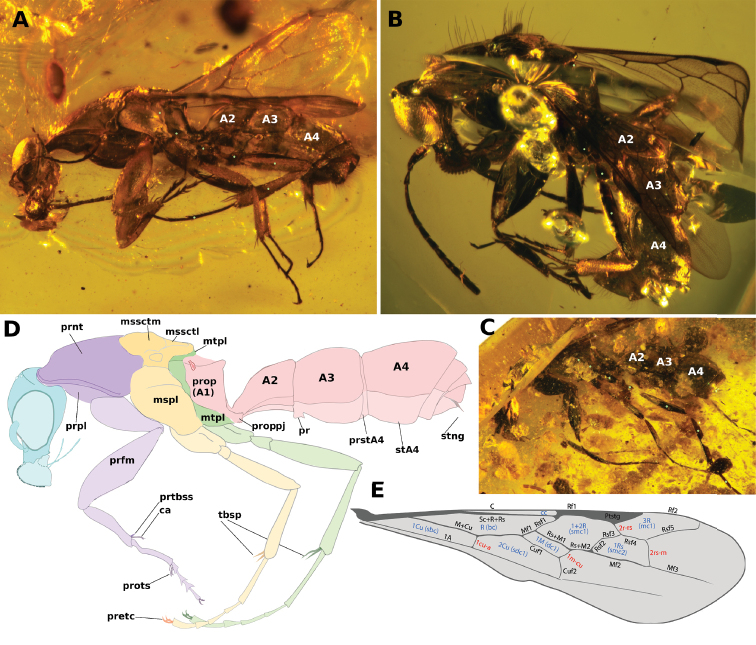
**A–C** †*Camelosphecia* spp. females examined by images. Specimens in **A** (BALBuTJ_36) and **C** (BALBuTJ_40) are likely conspecifics to †*C. fossor*. Specimen in **B** (BALBuTJ_38) is probably another species in the genus, not described in here (images by Phil Barden, used with permission) **D, E** are generalized schemes of †*Camelosphecia* habitus and forewing, respectively, as interpreted from all specimens examined. Metathoracic sclerites in **A–C** are indicated with green dots. In **D**, pedicel and flagellomeres are omitted for simplification. Abbreviations as in Figure 14, except for **prstA4**, presternite of A4; **prpl**, propleuron; **tbsp**, tibial spurs; **stA4**, sternite of A4; **stng**, sting. Wing venation abbreviations: **C**, costal vein; **Sc**, subcostal vein; **R**, radial vein; **Rs**, radial sector vein; **M**, medial vein; **Cu**, cubital vein, **A**, anal vein; +, indicates composite vein; **f**#, indicates free abscissa index; **2r-rs**, second radiosectoral crossvein; **2rs-m**, second sectoriomedial crossvein; **1m-cu**, first mediocubital crossvein; **1cu-a**, first cubitoanal crossvein; **R (bc)**, radial or “basal” cell; **1+2R (smc1)**, undifferentiated first and second distal radial cells or “first submarginal cell”; **3R (mc1)**, third distal radial or “first marginal” cell; **1Rs (smc2)**, first sectorial or “second submarginal” cell; **1M (dc1)**, first medial or “discal” cell; **1Cu (sbc)**, first cubital or “subbasal” cell; **2Cu (sdc1)**, second cubital or “first subdiscal” cell.

**Figure 16.**
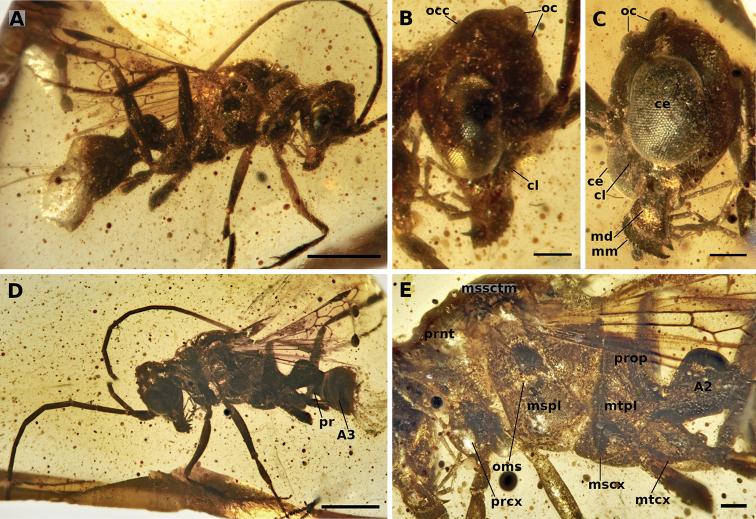
†*Camelosphecia venator* sp. nov. holotype (NIGP163574) **A, D** right and left profile view of body, respectively **B, C** right and left profile view of head, respectively **E** left mesosoma zooming. Abbreviations: **ce**, compound eye; **cl**, clypeus; **md**, mandible; **mm**, masticatory margins; **oc**, ocelli; **occ**, occipital carina; **A2–A3**; abdominal segments 2 and 3; **mscx**, mesocoxa; **mspl**, mesopleural area of mesopectus; **mssctm**, mesoscutum; **mtcx**, metacoxa; **mtpl**, metapleural area of mesopectus; **oms**, oblique mesopleural sulcus; **pr**, prora; **prcx**, procoxa; **prnt**, pronotum; **prop**, propodeum. Note that indication of clypeus in **C** is exactly at its margin as seen in profile, which is concave, and should not be confused with convex right compound eye margin, also indicated. Clypeal concave shape is better evidenced in figure **B**. Also in **C**, mm indicated is from the right mandible, while mandible indicated is that of the left side. Scale bars:1 mm (**A, D**); 0.2 mm (**B, C, E**).

Figs 1A , 2A , 13 , 14 , 15 , 16

##### Type species.

†*Camelosphecia fossor* sp. nov., by present designation.

##### Constituent species.

†*Cs. fossor* sp. nov., †*Cs. venator* sp. nov.

##### Diagnosis.

Identifiable as members of the †*Camelomecia* clade by the bowed mandibles with elongate masticatory margins, projecting clypeus, and absence of clypeal chaetae, frontal carinae, and facial projections, as outlined in the key above.

Both sexes specifically differentiated from †*Camelomecia* by: (1) the conspicuously-developed mandibular teeth on the masticatory margin (versus teeth present as mere crenulation or absent altogether); (2) fore wing 1cu-a crossvein distant proximally from divergence of free M and Cu by at least one of its own lengths (the phrase “markedly prefurcal” is used to describe this condition throughout this work; versus 1m-cu proximal to M+Cu split by less than one 1m-cu length, or 1m-cu usually at or distal to split, as observed in all known †*Camelomecia* and †Haidomyrmecinae, for example); and (3) crossvein 2m-cu absent (versus 2m-cu present or absent).

Females further differentiated from those of †*Camelomecia* as follows: (4) occipital carina of female extending to hypostoma (versus not); (5) compound eyes of female massively enlarged, filling entire lateral portion of head in profile view and rendering malar space virtually absent (versus compound eyes smaller, malar space well-defined); (6) teeth of masticatory mandibular margins conspicuously developed (versus present as crenulation or absent altogether); (7) disc (main central region) of labrum in the female bearing massive, long, thick chaetae (versus such chaetae absent); (8) anterior clypeal margin bidentate medially (versus margin edentate); (9) notauli on mesoscutum absent (versus present); (10) fore femora powerfully enlarged (versus weak and thin); (11) protarsomeres I and II margined with an array of differentiated psammochaetae (versus such chaetae absent); (12) posterolateral corners of propodeum armed (versus denticles absent or present); (13) abdominal poststernite IV short relative to posttergite.

Males, as so far known for both genera, are further differentiated from †*Camelomecia* in having: (14) eyes medially binocular, i.e., with clypeus nearly concave and compound eyes massively, medially bulging such that medial-most ommatidia of each eye are directed toward one another.

##### Etymology.

The root of the generic name, *camelo*-, is made in reference to †*Camelomecia*, the camel-faced ants; the second part of the name, -*sphecia* emphasizes the waspiness of these intermediate formicoids.

#### † Camelosphecia fossor sp. nov.

Taxon classificationAnimaliaHymenopteraFormicidae

E8E0DFB8-240A-52FD-B3A7-BB607D563FF9

http://zoobank.org/E6AD4D2B-89FA-4A5D-AFCE-5C1FC207F8A7

Figs 1A , 2A , 13 , 14 , 15

##### Holotype.

Myanmar, Kachin State: Hukawng Valley [**ANTWEB1038930**, deposited in **DZUP**].

##### Additional material examined.

Same as holotype [females: BALBuTJ-36, BALBuTJ-38, BALBuTJ-40 all in Janovitz collection]

##### Diagnosis.

Recognizable as Formicoidea and †*Camelosphecia* as defined above. Distinguished from †*C. venator* by features listed in that species’ diagnosis. In briefest, the most distinct features include massive compound eyes, huge and muscular profemora, and presence of psammochaetae on the protarsus.

##### Measurements.

**Female.**CL 0.76; HL 0.94; WH 1.08; CW 0.46; EL 0.41; MW 0.07; LW 0.06; A1L 0.21; A2L 0.11; A3L 0.22; PnW 0.66; PL 0.83; PnLm 1.05; MtW 0.66; MtL 0.41; MsW 0.37; MsL 0.32; WL 1.83; PtL 0.63.

##### Description.

**Female. *Head*.** Postgenal bridge elongate, thus head “prognathous”. Head posterior to clypeus strongly broadened (broader than long) and inflated, except for a pair of concavities lying over the posterolateral clypeal margin which accommodate the base of scapes. Mandibles cup-shaped, strongly bowed. Masticatory margin of mandibles with 13 teeth. Teeth, except the apical, truncate, gear-like; individual teeth short basally on masticatory margin and gradually increasing in length apically. Apical tooth (maybe apical and preapical) largest, pointed. Ventral surface of mandible without dense tuft of chaetae. Mandibles crossing apically, at full closure approximately half the masticatory margin would cross. Basolateral area of dorsal mandibular surface just beneath malar margin distinctly concave, contrasting to the remaining strongly convex, dome-shaped surface; the margin of this area just before meeting the cranium sinuous in profile view, appearing dentate. Labrum bilobed, its dorsum covered with ca. 24 long, stout chaetae with gently curved tips (chaetae somewhat shorter than 0.1 mm). Palps basally concealed by the labrum and mandibles, so that maxillary palpomeres total count is five or six and labial palpomeres are not visible. Maxillary palpomeres elongate. Clypeus having a basal section integrated with the cranium and an anteromedially projected over mandibles as a thin laminar platform, its maximum width ca. 0.4 × that of head. Clypeal platform having a pair of broad and low anterolateral lobes which curve into an anteromedial pair of close-set triangular teeth. In face view, considering the entire clypeus, the anterior clypeal margin bears two pairs of lobes and the medial pair of teeth, the lobes laterad corresponding to the actual anterolateral corners of the clypeus. Posterior to the platform, the remainder of the clypeus is rightly integrated into the cranium and confined to the anterior eighth of the head. The posterior margin of the clypeus is poorly marked medially, between the toruli, the margin slightly surpassing toruli anteriormost level, but not reaching their posteriormost level.

***Antennae.*** Torulus laterally directed; posteromedian portion of torular arch slightly enlarged and covering part of bulbus. Bulbus and bulbus neck coplanar and angled in relation to scape. Antennae 12-merous, not clubbed. Longest antennomeres are I (scape) and III, these two being subequal in length. Antennomere II (pedicel) is small and slightly inflated. Antennomere IV longer than V. Antennomeres V–XI more-or-less similar in size and shape. Apical antennomere not much longer than previous. Antennomeres dorsoventrally compressed (could be a taphonomic artifact). Frontal carinae absent. Eyes enormous, not bulging, length ca. 2 × the width in the full view of the eye; in full-face view, eye length ca. 0.7 × the length of head discounting clypeal projection. In full-face view, medial margin of compound eyes weakly concave, although not conspicuously notched. Ocelli relatively small and positioned high on dorsum of head, the lateral pair almost reaching the vertexal margin in full-face view. Vertexal margin slightly convex. Lateral margins almost entirely occupied by the compound eyes, except for anterior portions, which converge until meeting the outer margin of mandibles. Posterolateral and lateral occipital margin carinate, the margin poorly delimited posteromedially and anteromedially. Ventral surface of head flat to gently concave, with a slightly raised longitudinal median section corresponding in location with the postgenal ridge. Postgenal ridge with a small indentation at its posterior limit. Small, simple, suberect to recurved setae on dorsum of head, except for longer ones originating on the anterior edge of the clypeus and on the mandibles. Antennomeres II–XII covered on dense pubescence.

***Mesosoma.*** Pronotum elongate, distinctly bell-shaped in dorsal view; posteriormost region transversely constricted. Pronotum without anterior fringe of setae which is observed in †*Camelomecia*; anterior margin of pronotum, however, with distinct “beading” or rim and sulcus which delimit the anteriormost region. In profile view, posterodorsal portion extended posteriorly as a lobe, with the lobe situated very close to the tegula (nearly contacting); posterior margin just below lobe apparently concave; posteroventral margin lobate, not extending medially posterior to fore coxae, as would be expected for Apoidea. Mesonotum (mesoscutum + mesoscutellum) length in dorsal view subequal to length of pronotum. Mesoscutum > 1.5 × broader than long, more-or-less oval in shape. Axillae small. Scutoscutellar sulcus distinct and cross-ribbed, but not particularly deep. Mesoscutellum slightly longer than wide, tapering posterad and with an arched posterior margin. Mesopleuron bulging; oblique mesopleural sulcus present as a thin and poorly-marked line in the upper half of the mesopleural area posteriorly, separating the upper mesopleural area (erstwhile “anepisternum”) from the lower mesopleural area (erstwhile “katepisternum”); lower mesopleural area ca. 4 × longer than upper mesopleural area. Propodeal spiracle large, protruding from the cuticle, its opening slit and crescent-shaped and posteriorly oriented; spiracle positioned on the upper anterior region of the lateropropodeal surface. Lower metapleural area thin, delimited posteroventrally by carina. Upper metapleural area triangular, its margins approximately the same size. A small metanotal spiracle is apparently seen on the upper anterior corner of the left metapleural area. Propodeum with a subquadrate lamellate projection developed on the lower posterolateral mesosomal corner, projecting over the bases of the metacoxa and metasomal petiole. The anterior and posterior angles of the projection are sharply defined, and its dorsal surface is concave. Propodeum dorsal and declivous faces separated from the lateral face by a pair of ridges that are poorly marked anteriorly and strongly marked posteriorly, each reaching the metapleuropropodeal lamellate projection posteriorly; in the holotype, it appears that a pair of indentations on the upper portion of the propodeal ridges make the propodeum angled in profile view (at least on the left side of the specimen), whereas in the non-type specimens, these carinae toothed at the posterodorsal angle. Metapleural gland orifice absent, and there is no trace of a metapleural gland reservoir (bulla).

***Legs*.** Procoxa and profemur hypertrophied. Protibia bearing distally next to calcar a short, robust, spike-shaped chaeta. Calcar thin, curved, with bifid tip, without a distinct brush along its length, but having pubescence-like projections. Probasitarsal notch only gently concave, bearing the probasitarsal comb, but without any chaetae next to it. Probasitarsus and protarsomere II forming a specialized structure consisting of long, curved, somewhat bluntly tipped, psammochaetae. Probasitarsus anterior surface having two of such specialized sensilla trichodea apically, projecting over protarsomere II and protarsomere II bearing three of such sensilla trichodea on its anterior surface. The five psammochaetae are very close together and probably form a digging apparatus in analogy to extant fossorial Aculeata. In addition to the specialized chaetae, a pair of spike-shaped chaetae is also present apically on the posterior surface of probasitarsus as well as a tiny, peg-like chaeta apicomedially on the posterior surface. Protarsomere II has additionally a pair of small, spike-shaped chaetae on its posterior apical edge and an apicomedian, enlarged, blister-like, lobate chaeta on the posterior surface. Protarsomere II is short and slightly offset from the long axis of the probasitarsus. Protarsomeres III and IV each bearing two pairs of small, spike-shaped chaetae apicolaterally and with a lobate apicomedial chaeta in between them on the posterior surface, similar in shape but not as enlarged as that on protarsomere II. Spike-shaped chaetae and lobate chaeta apparently absent on protarsomere V. Pretarsal claws of proleg robust and curved, armed with a pair of teeth on their inner margins. Arolium slightly longer than half the length of claws. Mid and hind legs without any hypertrophied segment, although metatibiae apically clavate. Meso- and metatarsomeres I–IV similar in structure to protarsomeres III and IV, except for the apicomedian chaeta, which is not lobate and apparently rigid. These chaetae are also longer and more conspicuous on the mesotarsus than on the metatarsus. Paired mesotibial spurs present; spurs long and simple. Metatibial spurs not preserved, but from non-type material can be described as a pair of long and simple spurs similar but slightly longer than that on mesotibia.

***Metasoma*.** In total, three metasomal segments of holotype preserved, corresponding to abdominal segments II–IV. Petiole (abdominal II) massive in dorsal view; posttergite II anteriorly broad (approximately as broad as the width at midlength of the mesoscutellum), broadly inserting into the lower propodeal declivous surface (propodeal foramen wide). Laterotergite of segment II well-defined and dorsoventrally broad. Posttergite II mildly constricted posteriorly and consequently, constriction between pre- and posttergite III also not strong, therefore, the abdominal segment II has only mild petiolation. Poststernite II V-shaped in ventral view, its lateral margins carinate (could be a taphonomic artifact), tapering anteriorly until meeting and forming a small subpetiolar process (in profile largely obliterated by the metacoxae). Entire posttergite II and anterior portion of posttergite III laterally carinated. Anterior process on poststernite III, the prora, subrectangular; its anterior angle round and the posterior angle pointy and inclined ventrally. Abdominal segment IV, as determined from non-type specimens, with slight constriction corresponding to transverse sulci which delimit the pre- and post-sclerites of the tergum and sternum; lateral margins of tergum and sternum IV aligned for their whole length. Sclerites of abdominal segments V, VI, and VII telescoped internally, their tergal margins apparently overlap the sterna laterally.

***Wing venation*.** (Determined from non-type specimens.) Costal vein (C), subcostal-radial-radial-sector complex vein (Sc+R+Rs), and first free abscissa of the Radius (Rf1) present and tubular, enclosing costal cell. Pterostigma well-developed, situated near the apical third of the fore wing, but exact position difficult to ascertain. Rf distal to pterostigma present, meeting the free Radial Sector (Rsf) and enclosing third radial cell (3R1, or “first marginal cell”, 1MC). Cell 3R1 ca. 4 × as long proximodistally as wide anteroposteriorly; apex of cell rounded and considerably distant from apex of wing. The first free abscissa of the Radial Sector (Rsf1) splitting from Sc+R+Rs proximal to the pterostigma, but separated by ca. 1 of its lengths; Rsf1 directed posterobasally. The mediocubital complex vein (M+Cu) present; free Media (Mf) and Cubitus (Cuf) splitting near midlength of wing. First free abscissa of Media (Mf1) short, with a length subequal to that of the first cubitoanal crossvein (1cu-a). Rsf1 and (Mf1) meeting at a very oblique angle, nearly parallel; Radial-Sector-Media composite abscissa (Rs+M) tubular, directed posterodistally, and nearly orthogonal to main axis of Rsf1 and Mf1; Rs+M length subequal to that of Rsf1; split of Rs and M distal to anterior juncture of first mediocubital crossvein (1m-cu), thus Rs+M comprising two abscissae (Rs+M1, Rs+M2), and 1m-cu “prefurcal” in general aculeate terminology. 1m-cu short, subequal in length to Mf1. Rsf immediately distal to split of Rs+M, with apex of kink marked by flexion line, thus Rsf2 and Rsf3 defined; flexion line spectral, thus first radiosectoral crossvein (1r-rs) “absent”. Second radiosectoral crossvein (2r-rs) tubular, situated at approximately pterostigma midlength, directed slightly posterodistally, and short (length subequal to Mf1, 1cu-a). Rsf distal to 2r-rs divided into two remaining abscissae (Rsf4, Rsf5) by second sectoriomedial crossvein (2rs-m) [*note*: 1rs-m always absent in Hymenoptera due to fusion of Rsf and Mf which forms Rs+M]; Rsf4 longer than 2r-rs but shorter than Rf1, 2rs-m. Mf, distal to Rs+M, straight and divided into two abscissae by 2rs-m (Mf2, Mf3); Mf2 longer than Rs+M but shorter than Rsf5; Mf3 tubular but becoming spectral well before apex of wing. Two “submarginal cells” enclosed by tubular abscissae; third “submarginal cell” undefined due to absence of third sectoriomedial crossvein (3rs-m). First medial cell (1M, or “discal cell 1”) rhomboidal, ca. 4 × as long proximodistally as wide anteroposteriorly; Mf1 and 1m-cu parallel; Rs+M and first free cubital abscissa (Cuf1) parallel. Second medial cell (2M, or “*discal cell 2*”) undefined due to absence of the second mediocubital crossvein (2m-cu). The second free cubital abscissa (Cuf2) evenly and shallowly curved until its apex is directed posteriorly; Cuf2 apparently reaching first anal vein (1A); cubitus distal to Cuf nebulous to spectral and curved. 1cu-a situated considerably proximad M+Cu split, being distant by at least twice its length, hence 1cu-a “very prefurcal”. 1A tubular, although full extend uncertain. Hind wing venation not evaluated due to lack of appropriate preserved views.

***Preservation.* Holotype.** The body parts of the holotype that have suffered considerable distortion inside the amber matrix are the left procoxa, left mesopleuron and left mesocoxa, left and right metapleura and metacoxae, and the propodeum. Propodeal and metathoracic regions are considerably distorted, so much so that it is impossible to determine on which side the morphology has been better preserved. For example, on the right side, the propodeal spiracle is positioned at the same level of the mesocoxa in an anteroposterior axis, and the distance between the spiracle and the metapleural posteroventral corner is 0.43 mm. On the left side, it is positioned slightly anterior to the mesocoxa level and the distance between it and the metapleural corner is 0.32 mm. Missing body parts are: left protarsus; left mesotibia and mesotarsus; left metafemur, metatibia, and metabasitarsus; part of the right mesofemur and mesotibia, right metafemur, metatibia and metatarsus; entire abdominal segments IV–VII and part of abdominal segments II and III.

***Syninclusions.*** One nematoceran fly, which remains in the same amber piece with the holotype. Two staphylinid beetles, which were separated from the holotype in other amber pieces after the preparation (JCCamb00051 and JCCamb00052, both in JCMC).

##### Etymology.

The specific epithet emphasizes the digging adaptations of the species; the name is treated as a noun in apposition.

**Comments**. Four additional specimens of †*Camelosphecia*, three females (Fig. 15) and one male (Fig. 16), were studied based on images only. Among the females, two specimens (BALBuTJ_36 and BALBuTJ_40) are probably conspecifics to †*C. fossor* and one of them (BALBuTJ_38) probably represents another species. BALBuTJ_36 (Fig. 15A) is particularly interesting for its exceptional preservation. No significant differences were found between †*C. fossor* holotype and BALBuTJ_36, and the fossil was used to complete the description of †*C. fossor*, as most of the metasoma of the holotype was missing and its propodeum very distorted. BALBuTJ_40 (Fig. 15C) is a fossil difficult to interpret for containing a lot of debris and internal fractures around the inclusion. We doubtfully consider it conspecific to †*C. fossor*. A more thorough examination of the specimen can change this interpretation. BALBuTJ_38 almost certainly is a different species which we do not describe here. It differs from †*C. fossor* for having abundant thick, long and flexuous setae dorsally on mesosoma; unarmed, block-like propodeum; and an even thicker profemur.

#### † Camelosphecia venator sp. nov.

Taxon classificationAnimaliaHymenopteraFormicidae

AE4ECCCF-EB85-560E-807C-D139C4AC4C00

http://zoobank.org/00B043E6-1956-4EC6-A567-6186222B0280

Fig. 16

##### Holotype.

Myanmar, Kachin State: Hukawng Valley [**NIGP163574**, deposited in **NIGP].**

##### Diagnosis.

Identifiable as Formicoidea based on the definition given for the superfamily above. Associated with †*Camelosphecia* females by the multidentate mandibles, the shape of the clypeus, and the markedly prefurcal 1cu-a. †*Camelosphecia venator* differs substantially from †*C. fossor* and is undoubtedly a new species based on the following features: (1) “marginal cell” very short, area approximately equal to that of pterostigma; (2) 1m-cu “postfurcal”, or joining Mf distal to split of Rs+M; (3) 2r-rs joining Rsf proximal to 2r-rs; (4) “discal cell” wider; (5) “subdiscal cell” (enclosed by Cu, A, and 1cu-a) shorter; (6) petiolar node very well-defined, hump-like; and (7) prora (anteroventral keel of abdominal sternum III) shelf-like, strongly projecting. The male-based species differs from †*C. fossor* and †C. cf. fossor (BALBuTJ_38) by additional features which are expected due to sexual dimorphism, including having a distinct eye shape, shorter pronotum, twig-like profemora, and lack of the psammochaetae.

##### Measurements.

**Male.**CL 0.98; VBL 0.21; HL 1.34; EL 0.58; LW 0.16; A1L 0.20; A2L 0.09; A3L 0.39; PnL 0.48; PnLm 0.68; WL 1.76; WLa 1.62; PtL 0.42. (*Note*: due to preservation and orientation, could not measure HW, CW, MW, PnW, MtW, MtL, MsW, and MsL.)

##### Description.

**Male. *Head.*** Cranium “male-like” for Formicoidea, particularly stem Formicidae and taxa of the poneriine clade (i.e., the “poneroids” of Bolton 2003): Cranium more-or-less hypognathous despite elongate postgenal bridge; compound eyes bulging, medially emarginate; vertex (bearing ocelli) produced dorsally. Features differing from expectation: Mandibles distinctly multidentate, with eleven teeth as determined from the holotype; masticatory mandibular margin elongate; mandibles bowed, as observed in the female; cranial mandibular condyle small; clypeus reduced, concave, reminiscent of male Ponerini; compound eyes “binocular” in that anterior medialmost ommatidia with direct line of sight across the clypeus; antennal toruli close-set and dorsally directed (distinct from female); ocelli hypertrophied (suggesting nocturnal flight); occipital carina incomplete, possibly encircling occipital foramen but definitely not extending to mandibular base. Antenna 13-merous. Scape short, ca. 3–4 × as long as broad. Main body of pedicel approximately as broad as long. Flagellum elongate, each flagellomere several times longer than broad.

***Mesosoma.*** Pronotum short but muscular, with distinct bulge in profile view between “neck” and posterior “collar”; lateral face of mesopleuron broadly and deeply concave; concavity oriented dorsoventrally, apparently for reception of leg when fore leg completely retracted up to body; pronotum posterodorsally produced as lobe, lobe contacting fore wing tegulum; pronotum not forming ring posterior to fore coxae. Mesoscutum with deep and convergent notauli. Oblique mesopleural sulcus of mesopectus extending completely from anterior (“epicnemial”) margin to posterior (“mesepimeral”) margin. Mesothorax distinct laterally. Propodeum with dorsal and posterior faces curving into one another in profile view, apparently without distinct angular marking; posterolateral portion of propodeum, i.e., the area corresponding to the propodeal lobe, produced posteriorly, but not apparently in subrectangular form. Propodeal spiracle apparently situated high and anterior on segment, subtending metapleuron.

***Legs.*** Legs, overall, thin and without notable setal armament. Long setae not discernible. Protibial calcar apparently bifurcate apically. Mesotibia apparently with two ventroapical spurs, the anterior of which is thick compared to a seta and is barbirulate (*sensu*Bolton 2003). Metatibial spurs and tarsi not preserved in holotype.

***Metasoma.*** Abdominal segment II with distinct petiolar node which is strong and convex; anterolateral corners carinate; form of subpetiolar process uncertain. Helcium (articulatory portion of abdominal segment II) well-defined, axial (situated at approximately segment midheight), and broad dorsoventrally and lateromedially. Prora (keel of abdominal sternum II) robust and triangular in profile view.

***Wing venation.*** Veins tubular as in female †*Camelosphecia*. Differing as follows: 1Rsf situated ca. 2 × its length from pterostigma, nearly perpendicular to proximodistal length of wing; juncture of 1Rsf and Mf1 more distinctly angular; 1m-cu “postfurcal”, i.e., joining M distal to split of Rs+M; 2r-rs somewhat more proximal; “marginal cell” small, curve of posterior margin (as defined by Rsf) parallel to pterostigma; 2rs-m “prefurcal”, with anterior juncture proximal to 2r-rs; tubular portion of Mf distal to 2r-rs very short; “discal cell” pentagonal and less than 1.5 × as long proximodistally as broad anteroposteriorly; 1cu-a joining M+Cu ca. 1 × of its lengths proximal to split of Rs+M.

***Preservation.*** Amber matrix filled with uniformly distributed dark spheres. Metasoma from posterior portion of abdominal segment III, left meso- and meta-femora and distal segments, and right metatarsus removed due to specimen preparation. Hind wings not easily visible due to taphonomy. Specimen does not appear dehydrated or otherwise compressed or distorted.

##### Etymology.

The specific epithet suggests the likely predatory habits of the unknown female, while also highlighting the visual acuity of the male probably required for mate-seeking.

##### Comments.

We recognize that providing formal names to unassociated males risks inflating species-based biodiversity measures and runaway “parallel taxonomy” between sexes, as seen in various Dorylinae (e.g., *Neivamyrmex*) and Leptanillinae (e.g., *Leptanilla*). However, we are confident of the male-female pairing here due to the uniquely diagnostic mandibular conformation and markedly prefurcal 1cu-a crossvein. Moreover, the distinct wing venation and petiolar node of †*Cs. venator* provides both strong evidence of non-conspecificity with †*Cs. fossor*, and ample detail to associate unidentified females. For these reasons, we strongly recommend that any female which has a similar venational pattern and especially a nodiform petiole be considered conspecific with †*Cs. venator*, at least until further evidence accrues.

The marked reduction of the male’s cranium and pronotum coupled with hypertrophied or bulging eyes compared to the female strongly suggests specialized and sex-specific life histories. Among extant Formicidae, similarly enlarged eyes are often associated with nocturnal flights. At light sheets, such bug-eyed males are often observed *en masse*, without presence of conspecific females, suggesting either limited flights by females or the female-calling syndrome. Unfortunately, the genitalia of the unique specimen were lost during specimen preparation, thus the presence of copulatory specializations remains unknown. However, it is apparent from other male Formicoidea from Burmite and other ambers that a wide array of sexual modifications are known.

## Supplementary Material

XML Treatment for Formicoidea

XML Treatment for Camelosphecia

XML Treatment for Camelosphecia fossor

XML Treatment for Camelosphecia venator
